# A marginal estimate for the overall treatment effect on a survival outcome within the joint modeling framework

**DOI:** 10.1002/sim.8713

**Published:** 2020-08-24

**Authors:** Floor M. van Oudenhoven, Sophie H. N. Swinkels, Joseph G. Ibrahim, Dimitris Rizopoulos

**Affiliations:** ^1^ Department of Biostatistics Erasmus MC Rotterdam The Netherlands; ^2^ Danone Nutricia Research Utrecht The Netherlands; ^3^ Department of Biostatistics University of North Carolina Chapel Hill North Carolina USA

**Keywords:** joint models, marginal estimates, overall treatment effect, survival outcome.

## Abstract

Joint models for longitudinal and survival data are increasingly used and enjoy a wide range of application areas. In this article, we focus on the application of joint models on clinical trial data with special interest in the treatment effect on the survival outcome. Within a joint model, the estimated treatment effect on the survival outcome is an aggregate comprising the indirect treatment effect through the longitudinal outcome and the direct treatment effect on the survival outcome. This overall treatment effect is, however, conditional on random effects, and therefore has a subject‐specific interpretation. The conditional interpretation arises from the shared random effects between the longitudinal and survival process in combination with the nonlinear link function of the survival model. The overall treatment effect is, therefore, not valid for population‐based inference, which is the goal for most clinical trials. We propose a method to obtain a marginal estimate of the overall treatment effect on the survival outcome in a joint model. Additionally, we extend our proposal to allow for different parameterizations for the association between the longitudinal and survival outcome. The proposed method is demonstrated on data of a clinical study on the effect of synbiotic on the gut microbiota of cesarean delivered infants, where we estimate the marginal overall treatment effect on the risk of eczema or atopic dermatitis using longitudinal information on fecal bifidobacteria.

## 1 INTRODUCTION

Clinical trials frequently collect both longitudinal patient data, such as clinical biomarkers, in combination with survival data. Joint models for longitudinal and survival data allow a simultaneous analysis of these two types of data, thereby taking into account their interdependencies.^1^, ^2^ Joint models are an active area of biostatistics research and are due to recent developments in statistical software also increasingly applied in various medical fields, such as AIDS,^3^, ^4^ cancer,^5^ and Alzheimer.^6^, ^7^

This article is motivated by the application of joint models in clinical trial data with special interest in the treatment effect on the survival outcome. One such example that also formed the motivation for the research presented here is the Julius study.^8^ The Julius study is a randomized controlled study in cesarean born infants between June 2011 and April 2013 in Singapore and Thailand, intending to assess the effects of different infant formulas (standard, prebiotic, or synbiotic) on gut microbiota. The study showed positive effects in the synbiotic group compared to control, such as fast colonization by bifidobacteria from the first days after birth which contributes to emulate the gut physiological conditions observed in vaginally delivered infants, for more information see Chua et al.^8^ Although the study was not designed to measure any clinical endpoint as primary outcome, the incidence of skin disorders specifically eczema or atopic dermatitis was found to be lower in the synbiotic group. Previous research has found that delayed colonization by bifidobacteria is associated with eczema in cesarean born infants.^9^ Our goal here is to assess the overall treatment effect of synbiotic infant formula on the risk of eczema or atopic dermatitis, thereby taking into account the association with the longitudinal evolutions of fecal bifidobacteria using a joint model.

When only the treatment effect on the survival outcome is of interest, traditional survival methods such as the log‐rank test or the Cox model can be used. However, when interest is to gain understanding into the process of how a treatment affects the survival outcome via a biomarker pathway, joint models provide an attractive framework. In particular, using a joint model, we can distinguish the indirect treatment effect on the survival outcome through the longitudinal outcome, and the direct treatment effect on the survival outcome. Also, as a result, in the joint model, the overall treatment effect on the survival outcome is an aggregate comprising these indirect and direct treatment effects on the survival outcome. This was previously discussed by Ibrahim et al,^10^ and a sample size formula for the overall treatment effect was proposed by Chen et al,.^11^ Still, the topic has received little attention, and in the papers mentioned above, the treatment effect is defined as a constant effect over time. However, very often, in longitudinal clinical trials, the treatment effect is assumed to have a gradual effect over time rather than a constant effect. Modeling a time‐varying treatment effect on the longitudinal outcome in the indirect process of the joint model results in an overall treatment effect that has a time‐varying character. In this article, we start by providing a general definition of the time‐varying overall treatment effect. Moreover, the traditional joint model uses a mixed‐effects model for the longitudinal trajectories in combination with a Cox model for the survival outcome, with the association between the two processes partly induced by the shared random effects. Due to these shared random effects in combination with the non‐linear (i.e., exponential) link function of the Cox model, the overall treatment effect derived from a joint model has an interpretation conditional on the random effects, having a “subject‐specific” (SS) interpretation. The interpretation of the SS overall treatment effect differs from the marginal or “population‐averaged” treatment effect which has an interpretation for the individual rather than for the population. The SS overall treatment effect denotes the treatment effect for a typical or the *average* patient, that is, with random effects equal to zero. In contrast, the marginal overall treatment effect denotes the *average* treatment effect in the population. The difference between parameters with a SS and marginal interpretation has received a lot of attention within the mixed model literature, and it is generally recommended to decide between SS and marginal approaches, depending on the target of inference. For inference based on the individual level, for example, in personalized medicine, SS estimates are desirable, whereas for population‐based inference, marginal estimates are desirable. In the clinical trial setting, often marginal treatment effects are preferred, such that inferences can be generalized to the whole population, which is also the aim in our motivating clinical trial dataset.

Within the topic of clustered longitudinal data with non‐Gaussian outcomes, Heagerty et al^12^ proposed marginal multilevel models, which provides parameter estimates with a marginal interpretation while retaining the attractive features of likelihood‐based methods, for example, the ability to produce unbiased estimates when the data are missing at random. Additionally, Tsonaka et al^13^ extended these models to the framework of shared parameter models. Although these types of models provide attractive features, they can be computationally intensive. Hedeker et al^14^ proposed a method for the marginalization of regression parameters in mixed models for correlated binary outcomes with minimal additional computation. However, within the framework of joint models, the distinction between SS and marginal parameters is as yet unconsidered. The second important contribution of our work is, therefore, to propose a method for the marginal overall treatment effect on the survival outcome within the joint modeling framework, together with its standard error.

The article is organized as follows: In Section 2, the definition of the joint model, and the time‐varying overall treatment effect is given. Section 3 presents the proposed method for the marginal overall treatment effect and its standard error. In this section, we also propose an extension of our method to allow for different parameterizations for the association between the longitudinal and survival outcome. Section 4 shows the results for the proposed method on the motivating dataset. In Section 5, we evaluate the differences between the obtained marginal overall treatment effect and the SS overall treatment effect under different simulation scenarios.

## 2 THE OVERALL TREATMENT EFFECT IN A JOINT MODEL

### 2.1 Definition of a joint model

For subject *i*, (*i* = 1, … , *n*), let $T_{i}^{*}$ and *C*_*i*_, respectively, denote the true event time and the censoring time. Moreover, let $T_{i}=\min(T_{i}^{*},C_{i})$ be the observed survival time and $\delta_{i}=I(T_{i}^{*}\leq C_{i})$ be the event indicator, denoting $\delta_{i}=1$ if subject *i* experienced the event and 0 otherwise. For the longitudinal outcome, let *y*_*i*_(*t*) denote its value at time point *t* for the *i*th subject, being only observed at time points *t*_*ij*_ (*j* = 1, … , *n*_*i*_), and let *N* denote its total number of observations. Note that both the amount of measurements (*n*_*i*_) and the timing of measurements (*t*_*ij*_) can vary between subjects.

To describe the longitudinal evolutions of the percentage of bifidobacteria (from the total bacteria) over time, we use a mixed‐effects model, in particular we postulate:

$$\begin{array}{ll} y_{i}(t) & =\eta_{i}(t)+\epsilon_{i}(t)\;\\ & =x_{i}^{⊤}(t)\beta +z_{i}^{⊤}(t)b_{i}+\epsilon_{i}(t),\; \end{array}$$

where β denotes the vector for the fixed regression coefficients, ***b***_*i*_ denotes the vector of random effects, ***x***_*i*_(*t*) and ***z***_*i*_(*t*) are design vectors for the fixed and random effects, respectively, and $\epsilon_{i}(t)$ denotes the measurement error term for which we assume $\epsilon_{i}(t)\;∼𝒩(0,\sigma^{2})$. Furthermore, the random effects ***b***_*i*_ are assumed normally distributed with mean zero and covariance matrix $\sum_{b}$, being independent of $\epsilon_{i}(t)$. The term $\eta_{i}(t)$ denotes the true and unobserved value of the longitudinal outcome at any time *t*, which we associate with the risk of an event. To model the association, we use a relative risk model of the form:^2^, ^15^

(1) $$h_{i}(t|{ℋ}_{i}(t),w_{i})=h_{0}(t)\exp\{\gamma^{⊤}w_{i}+\alpha \eta_{i}(t)\},$$

where ${ℋ}_{i}(t)=\{\eta_{i}(u);0\leq u<t\}$ denotes the history of the true unobserved longitudinal process up to time point *t*, *h*_0_(*t*) denotes the baseline hazard, which we approximate using spline coefficients $\gamma_{h0}$, and ***w***_*i*_ is a design vector of baseline covariates with a corresponding vector of regression coefficients γ. The coefficient α quantifies the strength of the association between the true unobserved value of the longitudinal marker $\eta_{i}(t)$ and the risk of an event. Further, the full joint model parameter vector is denoted as ***ϑ*** = ${\left(\gamma_{h0}^{⊤},\beta^{⊤},\gamma^{⊤},\alpha ,\sigma^{2},\text{vech}{\left(\sum_{b}\right)}^{⊤}\right)}^{⊤}$, where $\text{vech}(\sum_{b})$ denotes the unique elements of the covariance matrix $\sum_{b}$.

Importantly, the design vector ***x***_*i*_(*t*) includes a treatment indicator *D*_*i*_, with *D*_*i*_ = 1 denoting treatment group and *D*_*i*_ = 0 denoting control group, and typically also its interaction with time *D*_*i*_ × *t* or other potential forms. Moreover, the design vector of baseline covariates ***w***_*i*_ generally also includes a treatment indicator *D*_*i*_. Note that, postulating a time‐varying treatment effect on the longitudinal outcome, for example, by using an interaction of treatment by time, will result in a time‐varying overall treatment effect.

### 2.2 Definition of the overall treatment effect

Based on the model formulation in the previous section, the overall treatment effect 𝒯(t) in a joint model takes the form of the time‐varying hazard ratio between the treatment and control group, with all other covariates held constant. For this, let ***W***^*D* = 1^ and ***W***^*D* = 0^ be two identical design matrices for the regression coefficients specified in the survival submodel, with as only difference that ***W***^*D* = 1^ belongs to the treatment group, and ***W***^*D* = 0^ belongs to the control group. In the same way, let ***X***^*D* = 1^ and ***X***^*D* = 0^ be two identical design matrices for the regression coefficients specified in the longitudinal submodel, with as only difference that ***X***^*D* = 1^ belongs to the treatment group, and ***X***^*D* = 0^ belongs to the control group. Then, the overall treatment takes the form of the following time‐varying hazard ratio:

(2) $$\begin{array}{ll} 𝒯(t) & =\frac{h^{D\;=\;1}(t)}{h^{D\;=\;0}(t)}=\frac{h_{0}(t)\exp[W^{D\;=\;1}\gamma +\alpha \{X^{D\;=\;1}(t)\beta +Z(t)b\}]}{h_{0}(t)\exp[W^{D\;=\;0}\gamma +\alpha \{X^{D\;=\;0}(t)\beta +Z(t)b\}]}\;\\ & \neq \exp[\bar{W}\gamma +\alpha \bar{X}(t)\beta ], \end{array}$$

in which $\bar{W}=W^{D=1}-W^{D=0}$ and $\bar{X}(t)=X^{D=1}(t)-X^{D=0}(t)$ denote the effects involving the treatment in the survival and longitudinal submodel, respectively. Within the joint modeling setting, due to the mixed‐effects models for the longitudinal trajectories, random effects are involved in the hazard function. These random effects do not cancel out in the calculation of the time‐varying hazard ratio due to the nonlinear (i.e., exponential) link function of the survival model. Omitting the random effects part in the calculation of Equation (2) will, therefore, not correspond to the marginal time‐varying hazard ratio but rather to the conditional hazard ratio controlling for, or holding constant, the random effects. Therefore, the overall treatment effect in Equation (2) could also be denoted by $𝒯{\left(t\right)}^{SS}$ to reflect that it has a SS interpretation, and this also carries for the previously defined regression coefficients in the survival submodel γ and α:

$$\begin{array}{ll} \gamma & \equiv \gamma^{SS}\;\\ \alpha & \equiv \alpha^{SS}.\; \end{array}$$

For the regression coefficients in the linear mixed‐effects model, β, it is known that they both have a SS and a marginal interpretation.^16^ However, in case the longitudinal submodel is extended to be a generalized linear mixed‐effects model, using a nonlinear link function such as the logit, this no longer holds.

As an example, consider a joint model in which the mixed‐effects submodel includes a linear time effect, a treatment effect, and its interaction with time, and the hazard function includes treatment as only baseline covariate:

$$\begin{array}{ll} y_{i}(t) & =\beta_{0}+\beta_{1}t+\beta_{2}{\text{trt}}_{i}+\beta_{3}({\text{trt}}_{i}\times t)+b_{i0}+b_{i1}t+\epsilon_{i}(t),\;\\ h_{i}(t|{ℋ}_{i}(t),w_{i}) & =h_{0}(t)\exp\{\gamma_{1}{\text{trt}}_{i}+\alpha \eta_{i}(t)\}.\; \end{array}$$

For this joint model, the SS overall treatment effect $𝒯{\left(t\right)}^{SS}$ denotes $\gamma_{1}^{SS}+\alpha^{SS}(\beta_{2}+\beta_{3}t)$. Note that if no interaction term of treatment by time is used, such as in the article by Ibrahim et al,^10^ the SS overall treatment effect reduces to $\gamma_{1}^{SS}+\alpha^{SS}\beta_{2}$.

Our goal is to derive an estimate for the overall treatment effect for the population of subjects instead of one that is conditional on subjects' effects. For this purpose, we define the marginal time‐varying overall treatment effect as:

(3) $$𝒯{\left(t\right)}^{M}=\frac{{\left\{h^{D=1}(t)\right\}}^{M}}{{\left\{h^{D=0}(t)\right\}}^{M}}=\exp\{\bar{W}\gamma^{M}+\alpha^{M}\bar{X}(t)\beta \},$$

in which no random effects are involved and for which we will need to derive the marginal estimates $\gamma^{M}$ and $\alpha^{M}$.

### 2.3 Weighted average of the time‐varying overall treatment effect

As we have noted, the treatment effect we obtain in our setting is a function of time and not a single estimate. However, in some instances, such a single estimate could be desired. For example, as the endpoint of a clinical trial or it could be of interest to compare treatment estimates using different methods or to make comparisons between different studies with the same treatment. Therefore, building on the approaches of Chen et al^17^ and Ibrahim et al,^18^ we also define a weighted average of the marginal overall treatment effect as:

$$\phi {\left(t_{0}\right)}^{M}=\exp\{\int_{0}^{t_{0}}\log[\frac{{\left\{h^{D=1}(t)\right\}}^{M}}{{\left\{h^{D=0}(t)\right\}}^{M}}]\Omega (t)dt\},$$

where Ω(t) is a weight function such that $\int_{0}^{t_{0}}\Omega (t)dt=1$ and *t*_0_ is the time point at the end of the trial. The weight function is allowed to depend on the underlying survival function, for example, to give more weight to periods when more subjects are at risk. In settings in which a treatment does not have an immediate effect, it could be desirable to put relatively less weight at the beginning of the trial. However, it should be mentioned that the choice of the weight function is a sensitive choice as it might be a bit arbitrary, and the inferences might change depending on its choice. Therefore, the choice of the weight function should be pre‐specified and in line with the inferential goals of the trial. Alternatively, the weight function can be set to 1.

## 3 MARGINALIZATION OF THE OVERALL TREATMENT EFFECT

### 3.1 Marginal parameters

Hedeker et al^14^ proposed a solution to obtain marginal estimates from their SS counterparts in mixed models for clustered binary outcomes, for example, in mixed logistic regression. In this work, we extend and adapt this method to calculate the marginal overall treatment effect as formulated under the joint modeling framework. The principle behind this approach is not to directly specify a marginal model, but to obtain the marginal parameters from the original SS parameters.

Suppose that we have fitted a joint model from which we obtained the estimates ${\hat{\gamma }}^{SS}$, ${\hat{\alpha }}^{SS}$, and $\hat{\beta }$. Using the relative risk model in Equation (1), the SS hazard for subject *i*, is defined as:

$$h_{i}(t|b)=h_{0}(t)\exp[w_{i}^{⊤}\gamma^{SS}+\alpha^{SS}\{x_{i}^{⊤}(t)\beta +z_{i}^{⊤}(t)b_{i}\}].$$

The regression parameters in this model have an SS interpretation. The marginal log hazard can be obtained by marginalizing over the random effects:

(4) $$\log h_{i}^{M}(t)=\log\{\frac{\int_{b}h_{i}(t|b)S_{i}(t|b)f(b)db}{\int_{b}S_{i}(t|b)f(b)db}\}.$$

The integral above does not have a closed‐form solution and needs to be approximated using numerical methods, such as adaptive Gaussian quadrature or Monte Carlo simulations. Here we use the latter to evaluate the integral as:

(5) $$\log{\hat{h_{i}}}^{M}(t)\approx \log\{\frac{\frac{1}{G}\sum_{g=1}^{G}h_{i}(t|b^{\left(g\right)})S_{i}(t|b^{\left(g\right)})}{\frac{1}{K}\sum_{k=1}^{K}S_{i}(t|b^{\left(k\right)})}\},$$

in which we use 15‐point Gauss‐Kronrod rule to approximate:

$$S_{i}(t|b)=\exp\{-\int_{0}^{T_{i}}h_{i}(u|b)du\}\approx \exp\{-P_{i}\overset{15}{\underset{u=1}{\sum }}\pi_{u}h_{i}(P_{i}s_{u}+P_{i}|b)\},$$

where *P*_*i*_ = *T*_*i*_/2 and $\pi_{u}$ and ***s***_*u*_ denote prespecified weights and abscissas, respectively.

The Monte Carlo integration is performed by, respectively, simulating *G* and *K* values for each of the random effects for each subject (taking *G* = *K* = 5000), using the multivariate distribution with covariance matrix ${\hat{\sum }}_{b}$ as estimated from the fitted joint model.

Our goal is to obtain the marginal parameters directly linked to the marginal overall treatment effect (3), which are also involved in the obtained marginal log hazard:

(6) $$\log h_{i}^{M}(t)=w_{h_{0}}^{⊤}(t)\gamma_{h_{0}}^{M}+w_{i}^{⊤}\gamma^{M}+\alpha^{M}\{x_{i}^{⊤}(t)\beta \}.$$

To simplify the notation, let $\log H^{M}$ represent the (*N* + *n*) × 1 vector of marginal log hazards that is obtained by stacking the log hazards of all subjects at all time points *t*. For the time points, we use both the (*N*) longitudinal time points, and the (*n*) event times, consistent with the counting process formulation of the extended Cox model. To solve the equation with respect to the marginal parameters of interest, we rewrite the equation above as the product between the design matrix and the marginal parameters of interest:

$$\log H^{M}=W_{h_{0}}\gamma_{h_{0}}^{M}+W\gamma^{M}+\alpha^{M}X\beta =\tilde{X}\theta^{M},$$

where $\tilde{X}=\left[\begin{array}{cc} W_{h_{0}}W & X\beta \end{array}\right]$and $\theta^{M}={\left\{{\left(\gamma_{h_{0}}^{M}\right)}^{⊤},{\left(\gamma^{M}\right)}^{⊤},\alpha^{M}\right\}}^{⊤}$, with $W_{h_{0}}$, ***W***, and ***X*** the design matrices, respectively, for the baseline hazard, the regression coefficients specified in the survival model, and the regression coefficients specified in the longitudinal model. Multiplying both sides of the equation by ${\left({\tilde{X}}^{⊤}\tilde{X}\right)}^{-1}{\tilde{X}}^{⊤}$, gives the relationship between the marginal parameters of interest and the marginal log hazard:

$$\theta^{M}={\left({\tilde{X}}^{⊤}\tilde{X}\right)}^{-1}{\tilde{X}}^{⊤}\log H^{M}.$$

Substituting $\log{Ĥ}_{i}^{M}$, obtained by Monte Carlo simulations, into the equation above yields ${\hat{\theta }}^{M}$, from which we obtain the marginal estimates ${\hat{\gamma }}^{M}$ and ${\hat{\alpha }}^{M}$. These estimates can be used in Equation (3) in order to obtain an estimate of the marginal overall treatment effect $\hat{𝒯}{\left(t\right)}^{M}$, and if desired also to obtain an estimate of the weighted overall treatment effect $\hat{\phi }{\left(t_{0}\right)}^{M}$.

### 3.2 Other parameterizations for the association structure

The relative risk model in Equation (1) denotes the standard joint model, assuming that the hazard of an event at any time *t* is related to the underlying value of the longitudinal outcome at the same time point. However, the underlying association structure between the longitudinal and the survival outcome could be of a more complex nature. Currently, the choice of an appropriate association structure for the two types of outcomes has been recognized as an important assumption within the topic of joint models and several alternative parameterizations have been proposed in literature.^15^, ^19^, ^20^ These alternative parameterizations can be seen as special cases of the following general notation of the relative risk model:

$$h_{i}(t|{ℋ}_{i}(t),w_{i})=h_{0}(t)\exp[\gamma^{⊤}w_{i}+f\{{ℋ}_{i}(t),\alpha \}],$$

where the interpretation of the previous defined parameters stays the same, and the function $f\{{ℋ}_{i}(t),\alpha \}$ specifies which characteristics of the longitudinal outcome are related with the hazard of an event. In this article, we will consider two common specifications other than the standard joint model, specifically:

$$\begin{array}{ll} f\{{ℋ}_{i}(t),\alpha \} & =\alpha \frac{d\eta_{i}(t)}{dt},\;\\ f\{{ℋ}_{i}(t),\alpha \} & =\alpha \int_{0}^{t}\eta_{i}(s)ds.\; \end{array}$$

For these parameterizations, the hazard of an event at time point *t* is, respectively, associated with the slope of the longitudinal outcome at and the cumulative value of the longitudinal outcome up to the same time point *t*. Naturally, the relative risk model could also be extended to include both the underlying value and the slope of the longitudinal outcome.

Also for these alternative specifications, random effects are involved in the hazard function which do not cancel out in the calculation of the time‐varying hazard ratio. The proposed method can be extended to allow for these alternative parameterizations, where in Equation (5) the hazard *h*_*i*_(*t* | *b*) involves the parameterization of choice. For example, if the slope of the longitudinal outcome is chosen as being related to the hazard of an event, then in Equation (5), we use $h_{i}(t|b)=h_{0}(t)\exp\{w_{i}^{⊤}\gamma^{SS}+\alpha^{SS}\frac{d\eta_{i}(t)}{dt}|b\}=h_{0}(t)\exp\{w_{i}^{⊤}\gamma^{SS}+\alpha^{SS}\{x_{i}^{*⊤}(t)\beta +z_{i}^{*⊤}(t)b_{i}\}$, where $x_{i}^{*}$ and $z_{i}^{*}$ are now the design vectors corresponding to the derivative of the longitudinal outcome. Additionally, we should then replace the design matrix $\tilde{X}$ by ${\tilde{X}}^{*}=\left[\begin{array}{cc} W_{h_{0}}W & X^{*}\beta \end{array}\right].$

For the proposed method to work, it is needed to have separate components in the longitudinal model for the fixed and random parts. The use of splines in the longitudinal outcome complicates the calculation of the derivative, possibly resulting in non‐linear functions with the fixed and random parts being tangled. For an example of this, involving the calculation of the derivative using B‐splines, we refer to Rizopoulos et al.^15^ In such a situation, the design vector corresponding to the derivative of the fixed part of the longitudinal outcome may alternatively be approximated numerically using the central difference approximation:

$$x_{i}^{*}(t)\approx \frac{x_{i}(t+\varepsilon )-x_{i}(t-\varepsilon )}{2\varepsilon },$$

where for *ε* we can take a value of 10^−3^ such that the approximation error is *O*(10^−6^). The design vector corresponding to the derivative of the random part $z_{i}^{*}(t)$ can be obtained in a similar manner.

Complications with nonlinearity may also be involved in the cumulative effect parameterization. In that case, the design vector corresponding to the integral of the fixed part of the longitudinal outcome $\int_{0}^{t}\eta_{i}(s)ds$ can be approximated numerically using the 15‐point Gauss‐Kronrod quadrature rule:

$$x_{i}^{*}(t)\approx \overset{15}{\underset{q=1}{\sum }}w_{q}x_{i}(t_{q})\beta ,$$

and similarly, for the design vector corresponding to the integral of the randompart $z_{i}^{*}(t)$.

### 3.3 Marginalized standard errors

Different methods are available for the computation of the standard errors. Hedeker et al,^14^ for example, used the delta method to estimate the standard errors associated with the marginal estimates. To apply the delta method here, requires the calculation of the first derivative of the marginalized parameters given by

$$\frac{\partial \theta^{M}}{\partial \vartheta }={\left({\tilde{X}}^{⊤}\tilde{X}\right)}^{-1}({\tilde{X}}^{⊤}\times \frac{\partial \log\{\frac{\int_{b}h(t|b)S(t|b)f(b)db}{\int_{b}S(t|b)f(b)db}\}}{\partial \vartheta }),$$

for which no closed‐form solution exists but which could be approximated numerically, for example, by Monte Carlo integration. Another common technique to approximate the standard errors is to use Bootstrapping.^21^ Applying bootstrap methods here would require estimating joint models numerous times, which is a computationally demanding task. Yet another possible approach to get the marginal standard errors is to use the second derivative of the observed data log‐likelihood, but this would require an expression of the marginal likelihood. Since we do not directly specify a marginal regression model, obtaining the marginal likelihood is not straightforward here.

Therefore, as an alternative we use Monte Carlo simulations to estimate the variability of the marginal overall treatment effect. Using Monte Carlo simulations is more straightforward compared to obtaining the marginal likelihood or applying the delta method, and more computationally efficient compared to Bootstrapping.

Suppose we have fitted a joint model and obtained estimates for the full SS parameter vector ${\hat{\vartheta }}^{SS}$, including ${\hat{\theta }}^{SS},$ and that the proposed method was used to obtain the marginal parameters $\gamma^{M}$ and $\alpha^{M}$. Then, to obtain the standard errors associated with these marginal parameters, we repeat the proposed method *l* = 1, … , *L* times, where each time instead of $\theta^{SS}$, we use $\theta^{SS*}$ based on a realization $\vartheta^{SS*}$ from the sampling distribution to calculate the marginal parameters. In particular, we use the following simulation scheme:

Step 1. Draw $\vartheta^{SS*}\;∼𝒩({\hat{\vartheta }}^{SS},\text{Var}({\hat{\vartheta }}^{SS})\;)$.

Step 2. Use Monte Carlo simulations to obtain the marginal log hazard based on $\theta^{SS*}$ of our realization $\vartheta^{SS*}$:

(7) $$\log\hat{h_{i}}{\left(t;\theta^{SS*}\right)}^{M}\approx \log\{\frac{\frac{1}{G}\sum_{g=1}^{G}h_{i}(t;\theta^{SS*}|b^{\left(g\right)})S_{i}(t;\theta^{SS*}|b^{\left(g\right)})}{\frac{1}{K}\sum_{k=1}^{K}S_{i}(t;\theta^{SS*}|b^{\left(k\right)})}\},$$

where $\theta^{SS*}={\left\{{\left(\gamma_{h0}^{SS*}\right)}^{⊤},{\left(\gamma^{SS*}\right)}^{⊤},\alpha^{SS*}\right\}}^{⊤}.$

Step 3. Compute

$${\hat{\theta }}^{M*}=(\overset{n}{\underset{i=1}{\sum }}{\tilde{X_{i}}}^{⊤}\tilde{X_{i}}){}^{-1}(\overset{n}{\underset{i=1}{\sum }}{\tilde{X_{i}}}^{⊤}\log\hat{h_{i}}{\left(t;\theta^{SS*}\right)}^{M}).$$

Step 4. Repeat steps 1‐3 *l* = 1, … , *L* times (e.g., taking *L* = 200), where L denotes the number of Monte Carlo samples.

The proposed procedure has Bayesian grounds because in step 1, we simulate from the approximate posterior distribution of the parameters to draw a new realization $\vartheta^{SS*}$. For this, we use arguments of standard asymptotic Bayesian theory^22^ and assume that the sample size of the sample, on which the joint model was fitted, is sufficiently large such that the posterior distribution of the parameters can be well approximated by $𝒩({\hat{\vartheta }}^{SS},\text{Var}({\hat{\vartheta }}^{SS})\;)$. This step accounts for the variability of the maximum likelihood estimates for the SS parameters. In step 2, we use Monte Carlo integration to obtain the marginal log hazard, as in Equation (5), and subsequently, in step 3, we use this estimate to compute the marginal parameter vector ${\hat{\theta }}^{M*}$. We repeat this *L* times which provides us multiple Monte Carlo samples of the marginal parameters $\gamma^{M}$ and $\alpha^{M}$, based on which we can compute the corresponding standard errors and the confidence intervals for the overall treatment effect, respectively, using the sample variance and the sample percentiles. We performed a simulation study to investigate the performance of the proposed procedure for the marginal standard errors, giving satisfactory results. More information and results are given in the supplemental material.

## 4 ANALYSIS OF THE JULIUS DATA

In this section, we illustrate the proposed method on the Julius data, briefly introduced in Section 1. In the Julius study, 153 infants delivered by C‐section were randomized to receive either standard formula (control), standard formula added with scGOS/IcFOS (prebiotic formula), or standard formula added with scGOS/IcFOS and *B. breve* M‐16V (synbiotic formula). Subjects received study products from birth until 16 weeks of age. Fecal samples were collected at day 3/5, week 2, week 4, week 8, week 12, and week 16, based on which the percentage of fecal bifidobacteria was determined. In this article, we focus on the modified‐intention‐to‐treat (mITT) population, consisting of all 129 subjects who provided at least one baseline and postbaseline fecal sample. During the study intervention period respectively, 10 subjects in the control (22.2 %) versus 3 subjects in the synbiotic group (6.7 %) experienced symptoms of eczema or atopic dermatitis. We estimate the marginal overall treatment effect on the risk of eczema or atopic dermatitis of synbiotic infant formula compared to control while using the longitudinal information on the percentages of fecal bifidobacteria.

Since a fast or delayed colonization by bifidobacteria at the beginning of life might affect the risk of an event at later time points, we choose to use a joint model in which the risk of an event at time *t* is associated with the cumulative value of the total fecal bifidobacteria up to time *t*. To describe the longitudinal evolutions, we used linear and quadratic time trends. To allow subjects to have different baseline levels and different time trends, we included random intercepts and slopes. However, note that the proposed method would also work for higher dimensions of the random effects structure. Furthermore, we corrected for the effect of site, which was either Singapore or Thailand. The mixed‐effects submodel for the percentages of fecal bifidobacteria is

$$y_{i}(t)=\beta_{0}+\beta_{1}{\text{trt}}_{i}+\beta_{2}t+\beta_{3}t^{2}+\beta_{4}({\text{trt}}_{i}\times t)+\beta_{5}({\text{trt}}_{i}\times t^{2})+\beta_{6}{\text{site}}_{i}+b_{i0}+b_{i1}t+\epsilon_{i}(t),$$

where trt_*i*_ denotes either control, prebiotic or standard formula. Furthermore, $\epsilon_{i}(t)\;∼𝒩(0,\sigma^{2})$ and $b_{i}∼𝒩(0,\sum_{b})$. For the relative risk submodel, we used a Cox model with treatment as the only baseline covariate

$$h_{i}(t|{ℋ}_{i}(t),w_{i})=h_{0}(t)\exp\{\gamma_{1}{\text{trt}}_{i}+\alpha \int_{0}^{t}\eta_{i}(s)ds\}.$$

**FIGURE 1:**
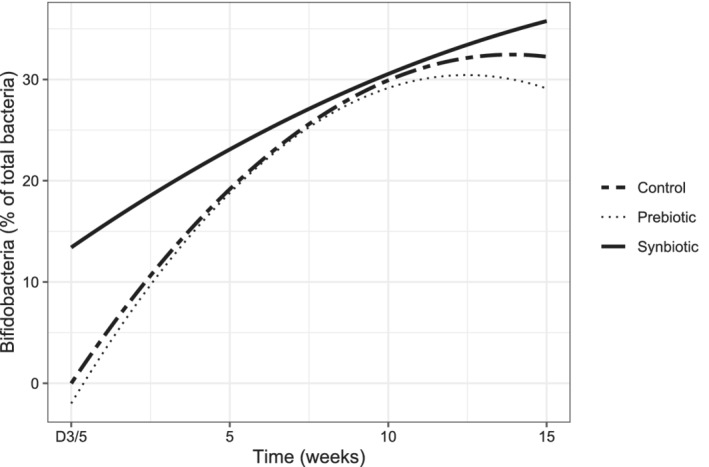
Estimated longitudinal profiles for bifidobacteria (% of total bacteria) for the different formula groups based on the postulated joint model

Figure 1 shows the estimated longitudinal profiles of the percentages of bifidobacteria for the different formula groups based on the joint model, from which we observe higher percentages of bifidobacteria in the synbiotic than in the control group in the first weeks after delivery. Note that the first sample was taken at day 3 or 5, after subjects received their first study product.

To estimate the overall treatment effect of synbiotic formula versus control based on the postulated joint model, we use the procedure described in Section 3.2 in order to deal with the cumulative effect parametrization. That is, in Equation (5), we use

$$\begin{array}{ll} h_{i}(t|b) & =h_{0}(t)\exp\{\gamma_{1}^{SS}trt_{i}+\alpha^{SS}\int_{0}^{t}\eta_{i}(s)ds\}\;\\ & =h_{0}(t)\exp[\gamma_{1}^{SS}trt_{i}+\alpha^{SS}\{\beta_{0}t+\beta_{1}({\text{trt}}_{i}\times t)+\beta_{2}t^{2}/2+\beta_{3}t^{3}/3+\beta_{4}({\text{trt}}_{i}\times t^{2}/2)\;\\ & \;+\beta_{5}({\text{trt}}_{i}\times t^{3}/3)+\beta_{6}({\text{site}}_{i}\times t)+b_{i0}t+b_{i1}t^{2}/2\}].\; \end{array}$$

This gives us estimates for the marginal parameters $\gamma_{1}^{M}$ and $\alpha^{M}$ from which we obtain an estimate of the marginal overall treatment effect given by

$$\begin{array}{ll} 𝒯{\left(t\right)}^{M} & =\exp\{\bar{W}\gamma^{M}+\alpha^{M}\int_{0}^{t}\bar{\eta }(s)ds\}\;\\ & =\exp[\gamma_{1}^{M}+\alpha^{M}\{\beta_{1}t+\beta_{4}t^{2}/2+\beta_{5}t^{3}/3\}],\; \end{array}$$

where $\bar{\eta }=\eta^{D=1}-\eta^{D=0}$, with *D* = 1 and *D* = 0, respectively, for the synbiotic and control group. Comparisons with the prebiotic formula are not of interest here.

**FIGURE 2:**
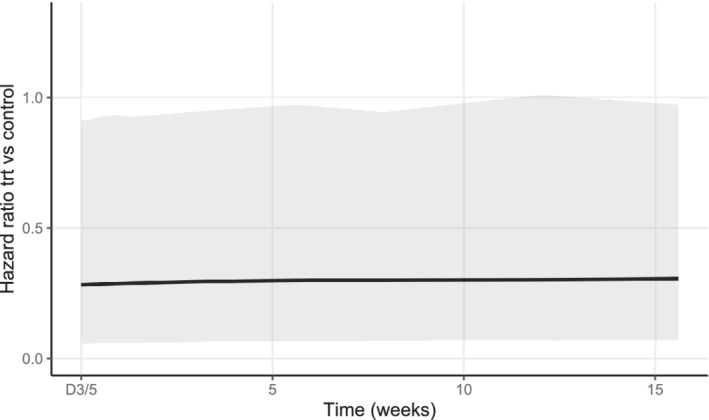
Estimated marginal time‐varying overall treatment effect $𝒯{\left(t\right)}^{M}$ of synbiotic infant formula compared to control on the risk of eczema or atopic dermatitis and corresponding 95% pointwise confidence interval

In Figure 2, we present the estimated marginal time‐varying overall treatment effect of synbiotic infant formula compared to control with pointwise 95% confidence intervals. We observe a significant overall treatment effect on the risk of eczema or atopic dermatitis from day 3/5 onward. Note that the overall treatment effect in this example does not have a strong time‐varying character. Furthermore, we observed that the obtained marginal time‐varying overall treatment is very similar to the SS overall treatment effect (not shown here). An added value of the joint model is that we can distinguish the direct treatment effect on the risk of eczema or atopic dermatitis from the indirect effect through the longitudinal outcome. For this specific application, we observed that the contribution of the direct treatment effect on the overall treatment is much larger than the contribution of the indirect treatment effect.

**FIGURE 3:**
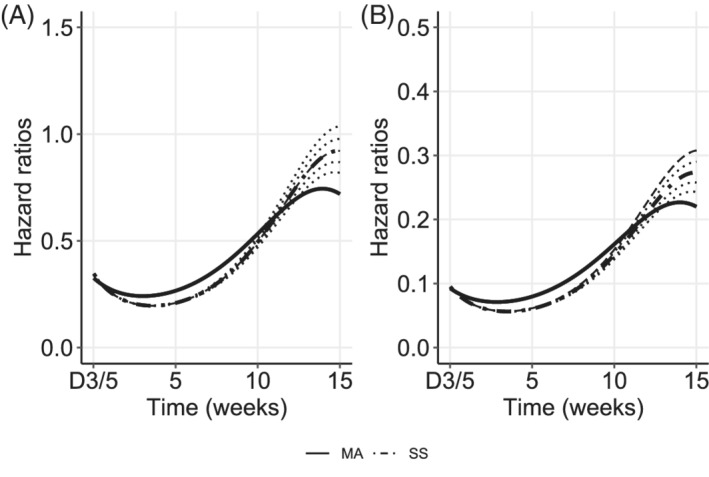
SS and marginal hazards in respectively the control group (A) and the treatment group (B) for the Julius dataset. SS hazards are displayed across a range of values for the random slope (dotted lines). The middle line (dot‐dashed, bold) corresponds to a random slope of 0. Note that the y‐axis scales are not the same in both panels

As part of our method, we obtain the marginal hazard. Although not being the quantity of primary interest, comparing the SS and marginal hazards in Figure 3 shows us an interesting pattern. In this figure, we present the SS and marginal hazards for respectively the control group (A) and the treatment group (B), with the SS hazards displayed across a range of values for the random slope (dotted lines). We see from this plot that the SS and marginal hazards are different, in a similar manner that marginal and SS probabilities curves differ in mixed logistic regression. For a graphical representation in mixed logistic regression, see, for example, Molenberghs et al.^23^ Note the strong connection between Figures 2 and 3. That is, the marginal overall treatment effect in Figure 2 is actually the ratio between the marginal hazard in the treatment group (panel B) versus the control group (panel A) in Figure 3. Furthermore, based on Figure 3, we can see that the estimated SS and marginal time‐varying overall treatment effects are similar in this example, as the differences between the SS and marginal hazard ratios are comparable for the control group and the treatment group. In Section 5, we investigate in which scenarios the SS and marginal overall treatment effect estimates are expected to be different and vice versa.

## 5 SIMULATION STUDY

### 5.1 Design

To evaluate the differences between the SS overall treatment effect $𝒯{\left(t\right)}^{SS}$ and the marginal overall treatment effect $𝒯{\left(t\right)}^{M}$, we conducted a simulation study. We investigated the effect of association parameters with varying magnitudes. This parameter was expected to be important as the impact of the random effects on the survival outcome depends on the magnitude of the association between the longitudinal and survival outcome. Therefore, larger differences between the SS and marginal parameters are expected for higher magnitudes of association. For this purpose, we simulated 300 datasets from a simple joint model, in which the longitudinal submodel included an intercept ($\beta_{0}$), a linear time effect ($\beta_{1}$), an interaction of treatment by time ($\beta_{2}$) and random intercepts and slopes (*b*_0_, *b*_1_). For the survival submodel, we used a Cox model with treatment as only baseline covariate ($\gamma_{1}$). For each dataset, we simulated 450 subjects with a maximum of eight equally spaced longitudinal measurements. Regarding the regression coefficients for the longitudinal and survival submodel, we, respectively, used $\beta_{0}=1.08$, $\beta_{1}=-0.08$, $\beta_{2}=0.10$, and $\gamma_{1}=1.48$. The baseline hazard function was simulated using B‐splines. For the censoring times, we used an exponential distribution with mean 18, resulting in censoring rates between 10% and 30%. We assumed right censoring.

In total, we considered four simulation scenarios, for which we used association parameters with an increasing magnitude, ranging from very low to very high: I) α = −0.01, II) α = −0.5, III) α = −1, and IV) α = −2. For more information on the simulations study, such as the covariance matrix for the random effects and the knots and spline coefficients used for the baseline hazard, we refer to the supplemental material.

### 5.2 Analysis and results

**FIGURE 4:**
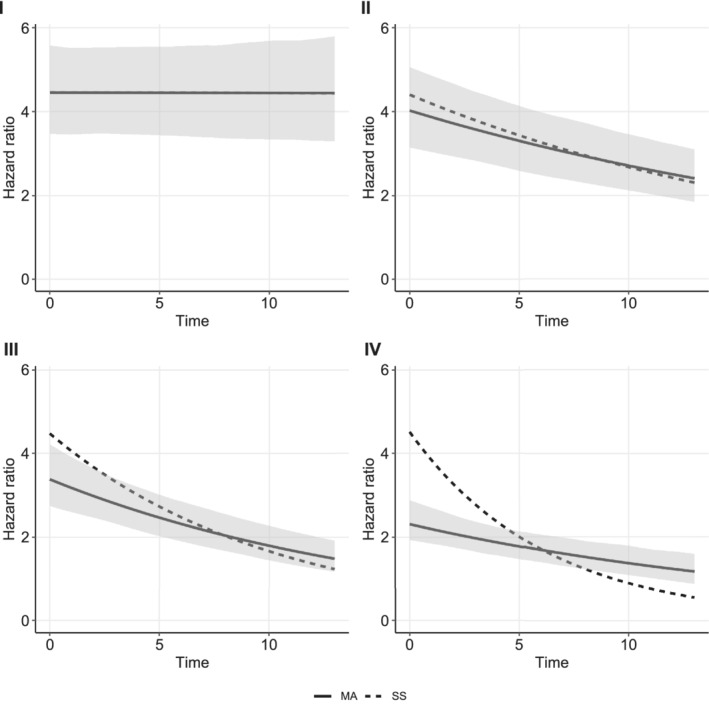
Average time‐varying SS overall treatment effect $𝒯{\left(t\right)}^{SS}$ and marginal overall treatment effect $𝒯{\left(t\right)}^{M}$ for simulation scenarios: I) α = −0.01, II) α = −0.5, III) α = −1.0, and IV)α=−2.0. Results are based on 300 simulated datasets. The gray band denotes the 95% percentille confidence interval based on the 300 obtained results for the marginal overall treatment effect

Each simulated dataset was analyzed with a joint model with the same specification as used in the simulation step. For each simulated dataset, we calculated the SS and marginal time‐varying overall treatment effects, from which the average effects for each scenario are shown in Figure 4. As expected, larger differences between the SS and marginal overall treatment effect are observed when the magnitude of the association parameter increases.

## 6 DISCUSSION

In this article, we considered the time‐varying character of the overall treatment effect in the joint model, which is a consequence of modeling a time‐varying treatment effect on the longitudinal outcome. Moreover, we have developed a method to obtain a marginal estimate for the overall treatment effect. This is relevant because often in clinical trials, marginal estimates are preferred, such that inferences can be generalized to the whole population. We also extended the proposed method to allow for different parameterizations for associating the longitudinal outcome with the hazard of an event.

We showed that in some cases, SS and marginal estimates for the overall treatment effect might be similar, such as in our motivating example. Whereas in other situations and in particular when the association parameter is of higher magnitude, their estimates may be (very) different.

Although the correctness of the results of the proposed method cannot be confirmed by a method that gives marginal estimates in this setting, the results of the simulation study were in line with our expectations. Moreover, a simulation study (not shown here) in which we used joint models with no random effects involved to simulate the data resulted, as anticipated, in SS and marginal effects that were identical.

In this article, we focused on normally distributed longitudinal outcomes using the linear mixed‐effects submodel. In the presented example, the percentage of bifidobacteria was a [0,100]‐bounded continuous longitudinal outcome, which is beta distributed rather than Gaussian. Although strictly speaking, this is a violation of the distributional assumption as fitted values may be outside the allowed 0‐100 range, from model diagnostics, we observed that the normal distributional assumption worked satisfactorily for the percentage of bifidobacteria.

Although the method was developed to obtain a marginal estimate of the overall treatment effect, it could also be used for other covariates, for example, to obtain the marginal overall effect gender or age on the survival outcome.

The proposed method has been implemented in the function marginal_coefs() in package **JM** (version 1.4‐8 available on GitHub) for the R programming language. An example of how to use this function can be found in the supplemental material.

## CONFLICT OF INTEREST

The authors Floor M. van Oudenhoven and Sophie H. N. Swinkels are employees of Danone Nutricia Research.

7

## Supporting information


**Data S1**: Supporting Information

## Data Availability

The data are proprietary information of Danone Nutricia Research. An example of how to use the function marginal_coefs() can be found in the supplemental material. The scripts of simulations are available on request.
