# Supplementary material for: A marginal estimate for the overall treatment effect on a survival outcome within the joint modeling framework
Source: Stat Med. 2020 Aug 24;39(28):4120–32. doi: 10.1002/sim.8713 (PMC7674249; doi:10.1002/sim.8713)
Supplement: Supplementary file 1 — Data S1: Supporting Information [file SIM-39-4120-s001.pdf]

Supporting information for A Marginal Estimate for the Overall Treatment Effect on a Survival Outcome within the Joint Modeling Framework

Floor M. van Oudenhoven[1,2], Sophie H. N. Swinkels[2], Joseph G. Ibrahim[3] and Dimitris Rizopoulos[1]

1. Department of Biostatistics, Erasmus MC, Rotterdam, The Netherlands
2. Danone Nutricia Research, Utrecht, The Netherlands
3. Department of Biostatistics, University of North Carolina, Chapel Hill, USA

1 Simulation Studies

1.1 Simulation Study settings

The data in the simulation study presented in Section 5 of the main paper have been simulated under the following joint model:

$$\begin{cases} y_i(t) = \eta_i(t) + \epsilon_i(t), \\ \eta_i(t) = \beta_0 + \beta_1 t + \beta_2(t \times trt_i) + b_{i0} + b_{i1}t, \\ \epsilon_i(t) \sim \mathcal{N}(0, \sigma^2), \\ \quad \boldsymbol{b}_i \sim \mathcal{N}(0, \Sigma_b), \\ \\ h_i(t) = h_0(t)\exp\{\gamma_1 trt_i + \alpha\eta_i(t)\}, \\ h_0(t) = \gamma_{h_0} B_{h_0}(t, 9). \end{cases}$$

We used the following simulating settings:

- For each of the 450 subjects longitudinal measurements were planned to be taken at 8 equally spaced visits divided over a follow-up period of 18 years;
- Fixed effects longitudinal model: $\beta_0 = 1.08$, $\beta_1 = -0.08$ and $\beta_2 = 0.10$;
- Random effects covariance matrix:
  $\Sigma_b = \begin{bmatrix} 0.75 & 0.002 \\ 0.002 & 0.001 \end{bmatrix}$
- Measurement error standard deviation: $\sigma = 0.25$;

- For the baseline hazard we used boundary knots at 0.002, 0.002, 0.002, 0.002, 7.367, 12.200, 13.200, 15.157, 17.410, 21.400, 21.400, 21.400 and 21.400 and spline parameters γ_{h01} = -3.012, γ_{h02} = -0.256, γ_{h03} = -3.093, γ_{h04} =-0.340, γ_{h05} =-1.936, γ_{h06} =-1.096, γ_{h07} =-1.871, γ_{h08} =-0.186 and γ_{h09} =-6.640;
- Baseline covariates survival model: $\gamma_1 = 1.48$;
- Association parameters:
  Scenario I: $\alpha = -0.01$
  Scenario II: $\alpha = -0.5$
  Scenario III: $\alpha = -1$
  Scenario IV: $\alpha = -2$
- The censoring mechanism was based on an exponential distribution with mean 18;
- For the Monte Carlo integration we used $M = 5000$.

1.2 Investigating the performance for the proposed procedure for the marginalized standard errors

Under the setting of the simulation study we investigated the performance of the proposed procedure for the marginalized standard errors. We simulated 500 joint models based on which we obtained 500 estimates of $\hat{\theta}^M$ for which we calculated their actual standard errors SE($\hat{\theta}^M$). We compared these estimates with the marginalized standard errors based on the proposed procedure for a joint model, simulated under simulation scenario II and III.

Table 1: Estimated marginalized standard errors using the proposed procedure versus actual standard errors of the marginal estimates.

	Estimated SEs	Actual SEs
$\alpha = -0.5$		
γ^M	0.112	0.115
α^M	0.039	0.039
$\alpha = -1$		
γ^M	0.104	0.104
α^M	0.021	0.038

2 The function marginal_coefs

As an example, we show how to use the function `marginal_coefs()` on the PBC data (freely available in R).

```
## Load packages
library(nlme)
library(survival)
library(JM) # contains the function marginal_coefs
```

```
library(plyr)
library(MASS)

# Fit joint model
lmeFit.pbc <- lme(log(serBilir) ~ year + drug:year, random = ~ year|id,
                  data = pbc2)
coxFit.pbc <- coxph(Surv(years, status2) ~ drug, data = pbc2.id, x = TRUE)
jointFit.pbc <- jointModel(lmeFit.pbc, coxFit.pbc, timeVar = "year",
                           method = "spline-PH-aGH")
summary(jointFit.pbc)

# Obtain marginal and subject-specific coefficients
results.marg <- marginal_coefs(jointFit.pbc)
result.marg

$ss_coefs
          bs1             bs2             bs3             bs4             bs5
 -4.061918981    -4.834203946    -4.030207356    -4.668552924    -4.475463306
          bs6             bs7             bs8             bs9    drugD-penicil
 -4.508950954    -2.282596790    -8.401054653    -3.144450534     0.073669295
        alpha      (Intercept)            year year:drugD-penicil
  1.242907770      0.491906733     0.183572148     0.003413077

$marg_coefs

          bs1             bs2             bs3             bs4             bs5
 -4.105249424    -5.115922941    -4.865685696    -6.391888739    -7.092577236
          bs6             bs7             bs8             bs9    drugD-penicil
 -7.997297358    -8.138847550   -14.778521056   -10.367305238     0.016259596
        alpha      (Intercept)            year year:drugD-penicil
  2.872717747      0.491906733     0.183572148     0.003413077

# Compare marginal and subject-specific overall treatment effect over time
max.t <- round(max(jointFit.pbc$data$year),0)
t <- seq(0, max.t, by = 0.01)
TE_MA <- exp(results.marg$marg_coefs[10] + results.marg$marg_coefs[11] *
             results.marg$marg_coefs[14] * t)
TE_SS   <- exp(results.marg$ss_coefs[10] + results.marg$ss_coefs[11] *
               results.marg$ss_coefs[14] * t)
```
